# Protein phosphatase 1 regulatory subunit 1A regulates cell cycle progression in Ewing sarcoma

**DOI:** 10.18632/oncotarget.27571

**Published:** 2020-05-12

**Authors:** Wen Luo, Changxin Xu, Sarah Phillips, Aliza Gardenswartz, Jeremy M. Rosenblum, Janet Ayello, Stephen L. Lessnick, Huai-Xiang Hao, Mitchell S. Cairo

**Affiliations:** ^1^ Department of Pediatrics, New York Medical College, Valhalla, NY, USA; ^2^ Department of Pathology, New York Medical College, Valhalla, NY, USA; ^3^ James J. Peters Veterans Affairs Medical Center, Bronx, NY, USA; ^4^ Department of Medicine, New York Medical College, Valhalla, NY, USA; ^5^ Nationwide Children’s Hospital, Columbus, OH, USA; ^6^ Novartis Institutes for BioMedical Research, Cambridge, MA, USA; ^7^ Department of Immunology and Microbiology, New York Medical College, Valhalla, NY, USA; ^8^ Department of Cell Biology and Anatomy, New York Medical College, Valhalla, NY, USA

**Keywords:** PPP1R1A, cell cycle control, IGF-1R, metastasis, Ewing sarcoma

## Abstract

Ewing sarcoma (ES) is a malignant pediatric bone and soft tissue tumor. Patients with metastatic ES have a dismal outcome which has not been improved in decades. The major challenge in the treatment of metastatic ES is the lack of specific targets and rational combinatorial therapy. We recently found that protein phosphatase 1 regulatory subunit 1A (PPP1R1A) is specifically highly expressed in ES and promotes tumor growth and metastasis in ES. In the current investigation, we show that PPP1R1A regulates ES cell cycle progression in G1/S phase by down-regulating cell cycle inhibitors p21^Cip1^ and p27^Kip1^, which leads to retinoblastoma (Rb) protein hyperphosphorylation. In addition, we show that PPP1R1A promotes normal transcription of histone genes during cell cycle progression. Importantly, we demonstrate a synergistic/additive effect of the combinatorial therapy of PPP1R1A and insulin-like growth factor 1 receptor (IGF-1R) inhibition on decreasing ES cell proliferation and migration *in vitro* and limiting xenograft tumor growth and metastasis *in vivo*. Taken together, our findings suggest a role of PPP1R1A as an ES specific cell cycle modulator and that simultaneous targeting of PPP1R1A and IGF-1R pathways is a promising specific and effective strategy to treat both primary and metastatic ES.

## INTRODUCTION

Ewing sarcoma (ES) is an aggressive bone and soft tissue tumor in children, adolescents, and young adults. Metastasis at diagnosis is present in approximately one-fourth of all patients and is associated with poor prognosis (5-year overall survival of ≤ 30%) [1]. Despite advances in surgery, radiation, chemotherapy, and megatherapy, the dismal outcome of these high risk ES patients has not improved in the past 30 years. Novel specific therapeutic strategies are urgently needed.

ES is characterized by the expression of chimeric fusions of EWS and ETS family transcription factors, mostly EWS/FLI, as a consequence of chromosomal translocation [2]. EWS/FLI acts as an aberrant transcription factor and master regulator of ES development by dominating dysregulation of its downstream targets in ES initiation and progression. We recently identified *protein phosphatase 1 regulatory subunit 1A* (PPP1R1A), a gene encoding a potent *protein phosphatase 1* (PP1) inhibitor, as one of the significantly upregulated EWS/FLI core targets. More importantly, we found that PPP1R1A regulates ES tumorigenesis and metastasis via the protein kinase A (PKA)/PPP1R1A/PP1 pathway. PPP1R1A depletion or a small molecule inhibitor of the PKA/PPP1R1A/PP1 cascade decreased tumor growth and metastasis in an ES orthotopic xenograft mouse model [3]. In the current study, we report that PPP1R1A plays an additional role as an ES specific cell cycle modulator.

Cell cycle progression is a process tightly regulated by both positive (CDKs and cyclins) [4] and negative regulators (INK4 and Cip/Kip families) [5]. Mutations in the genes involved in cell cycle regulation often underlie uncontrolled proliferation and oncogenesis. However, how the cell cycle is dysregulated in ES and whether EWS/FLI contributes to uncontrolled cell proliferation in ES remains unclear. Similar to other pediatric solid tumors, ES has a relatively quiet genome with few recurrent somatic mutations. Only a fraction of ES tumors contain genetic alterations, mostly mutations in *TP53* and *CDKN2A*, found to facilitate dysregulation of the cell cycle. Recently, one of the positive cell cycle regulators, *CDK4,* was identified as an Ewing-selective dependency gene and CDK4/6 inhibitors showed promising activity in ES models [6]. However, mutations affecting CDK4 and other cell cycle positive regulators such as cyclins occur much less frequently in ES [7]. Consequently, it is possible that inactivation of cell cycle negative regulators is the mechanism underlying ES development. In support of this concept, loss of p21^Cip1^ and p27^Kip1^ expression has been shown in ES primary tumor samples [8, 9]. In addition, it has been suggested that *p21*^Cip1^ may be a direct target of EWS/FLI, although the DNA binding site has not been identified [10]. In this study we show that the EWS/FLI upregulated target, PPP1R1A, inhibits negative cell cycle regulators p21^Cip1^ and p27^Kip1^ to promote cell cycle progression. It was previously shown that insulin-like growth factor 1 receptor (IGF-1R) inhibition enhanced the effect of CDK4/6 inhibitors on suppression of ES cell proliferation and tumor formation [11]. We found that combinatorial therapy of PPP1R1A inhibition with an IGF-1R inhibitor was more effective not only in limiting primary xenograft tumor growth, but also in decreasing lung metastasis, demonstrating a promising specific strategy to treat both primary and metastatic ES.

## RESULTS

### PPP1R1A depletion results in cell cycle arrest in G1 to S phase transition

**Figure 1:**
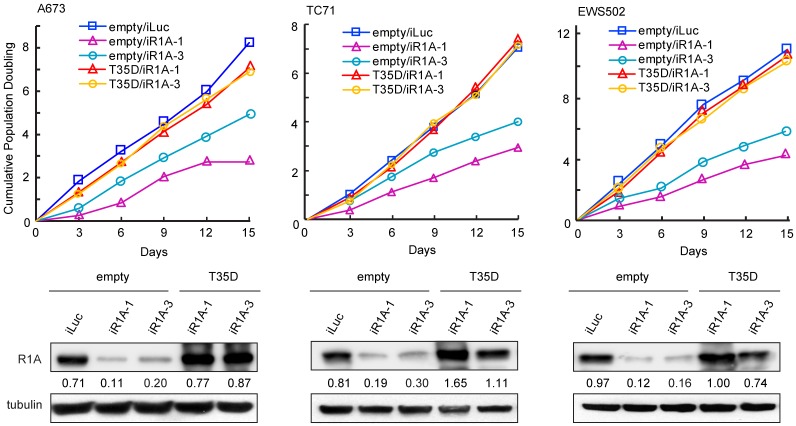
Depletion of PPP1R1A inhibits ES cell proliferation. PPP1R1A knockdown in ES cells results in reduced cell growth which can be rescued by expression of constitutively active PPP1R1A (T35D). Cumulative population doubling rate of control (iLuc) or PPP1R1A (iR1A-1 or -3) knockdown cells rescued by empty vector or T35D over 15 days (*upper*) and PPP1R1A protein levels in these cells (*lower*) are shown.

**Figure 2:**
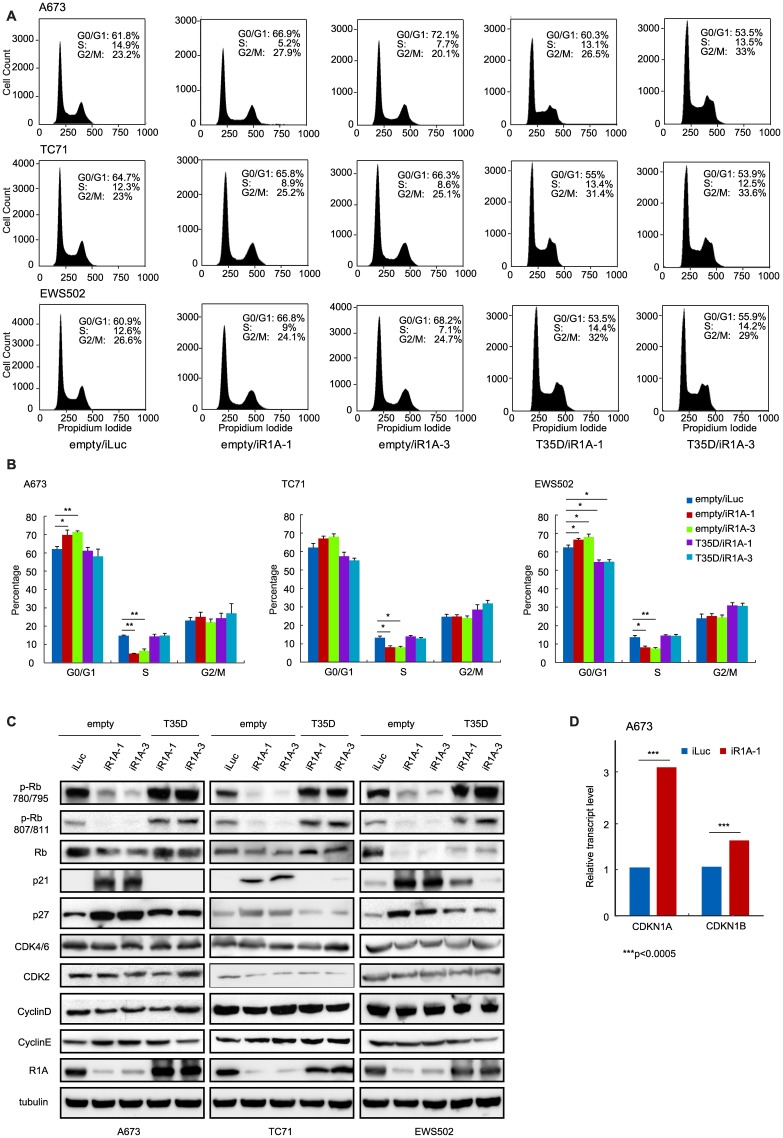
PPP1R1A controls G1 to S transition in ES cell cycle. (**A** and **B**) PPP1R1A depletion induces blockage of cell cycle at G1 to S transition phase which can be released by expression of T35D. ES A673, TC71, and EWS502 cells with control (iLuc) or PPP1R1A (iR1A-1 or -3) knockdown and rescued by empty or T35D were subject to cell cycle analyses by propidium iodide staining followed by flow cytometry. Quantification of percentage of cell population underwent each cell cycle phase is shown in B. (**C** and **D**) Low levels of PPP1R1A (empty/iR1A-1 or empty/iR1A-3) results in hypophosphorylation while high levels of PPP1R1A (empty/iLuc, T35D/iR1A-1, T35D/iR1A-3) induces hyperphosphorylation of Rb protein at sites 780/795 and 807/811. G1 cell cycle inhibitors p21^Cip1^ and p27^Kip1^ are downregulated by PPP1R1A at protein and transcript levels as evidenced by western blotting (C) and RNA-seq (D) analyses. The stripes over the images of A673 CDK2 and CyclinD in C are scratches on the X-ray film. *CDKN1A* and *CDKN1B* are genes encoding p21^Cip1^ and P27^Kip1^, respectively. ^***^multiple testing adjusted *p* < 0.0005.

In our previous report, we demonstrated that PPP1R1A promotes tumorigenesis and metastasis in ES via the PKA/PPP1R1A/PP1 pathway [3]. In the current study, we seek to further define the function of PPP1R1A and improve our understanding of the underlying molecular mechanism (s) in order to discover more potential effective therapeutic strategies for ES. We identified that knockdown of PPP1R1A by shRNA (iR1A-1 and -3) resulted in marked decrease in cell proliferation in multiple ES cell lines compared to control knockdown cells (iLuc) (Figure 1). This effect is specific to PPP1R1A but not off-target, because a constitutively active PPP1R1A (T35D) successfully rescued the decrease of cell growth induced by depletion of PPP1R1A (Figure 1). We observed a similar effect on cell growth with a CRISPR-Cas9 mediated knockout of PPP1R1A (R1A KO3) (Supplementary Figure 1). We further investigated the effect of PPP1R1A depletion on the cell cycle using flow cytometry DNA content analysis. Compared to the control knockdown cells, PPP1R1A knockdown in multiple ES cell lines markedly decreased the proportion of cells in the S phase. Again, the knockdown effect on cell cycle progression could be rescued by the expression of T35D (Figure 2A, 2B). These data indicate that PPP1R1A depletion leads to cell cycle arrest predominantly in the G1 to S phase transition.

### PPP1R1A regulates Rb phosphorylation

The tumor suppressor Rb protein plays a key role in the regulation of cell cycle, mainly as a G1 checkpoint, blocking S phase entry and cell growth. Dephosphorylation of Rb blocks cell cycle progression while phosphorylation of Rb releases cell cycle arrest in G1 phase. We proceeded to examine the correlation between phosphorylation status of Rb and depletion of PPP1R1A in multiple ES cell lines using antibodies specific for phosphorylated Rb at residues 780/795 and 807/811 which are phosphorylated by CDK4/6 and CDK2 during G1 phase, respectively. As shown in Figure 2C, Rb was hyperphosphorylated at residues 780/795 and 807/811 in cells with high PPP1R1A levels (iLuc/empty or iR1A-1/T35D or iR1A-3/T35D) and hypophosphorylated in PPP1R1A knockdown (iR1A-1/empty or iR1A-3/empty) cells (Figure 2C and Supplementary File 1). We also observed decrease in total Rb level in the PPP1R1A knockdown cells compared to that in the control knockdown or the knockdown/rescue cells. This change is likely due to phosphorylation-induced changes in Rb protein stability [12]. These findings suggest that PPP1R1A up-regulates Rb phosphorylation by CDKs.

### PPP1R1A downregulates cell cycle inhibitors p21^Cip1^ and p27^Kip1^

The observation that depletion of PPP1R1A results in activation of Rb prompted us to investigate the G1 phase regulatory proteins upstream of Rb, including CDK4/6, CDK2, cyclin D, cyclin E, CDK inhibitors p16^Ink4a^, p21^Cip1^, p27^Kip1^, and p57^Kip2^. We found that the levels of CDKs and cyclins had minimum changes, suggesting that expression of these G1 regulatory proteins were not affected by PPP1R1A. However, we found that the level of one of the CDK inhibitors, p21^Cip1^, was markedly increased in PPP1R1A depleted cells (iR1A-1/empty and -3/empty). A milder increase in the level of p27^Kip1^, another CDK inhibitor, was also observed (Figure 2C and Supplementary File 1). The changes of these cell cycle regulators in protein levels were correlated with the changes in RNA level. As shown by the RNA-seq data from control (iLuc) or PPP1R1A knockdown (iR1A-1) A673 cells, PPP1R1A down-regulates transcription of genes encoding p21^Cip1^ (CDKN1A) and p27^Kip1^ (CDKN1B) (Figure 2D). These findings suggest that PPP1R1A down-regulates cell cycle inhibitors p21^Cip1^ and p27^Kip1^ in protein and RNA levels which in turn leads to Rb hyperphosphorylation and release of the cell cycle block at G1 phase in ES cells.

### PPP1R1A controls transcription of replication-dependent histone genes

Using the Database for Annotation, Visualization and Integrated Discovery (DAVID) functional annotation analysis of the RNA-seq data we generated from control (iLuc) or PPP1R1A knockdown (iR1A-1) A673 cells, we identified functional classes that were enriched in the PPP1R1A regulated genes and found that nucleosome core is one of the significantly enriched terms in the down-regulated gene list (Supplementary Figure 2A). A closer inspection of the genes categorized in this functional class that are significantly down-regulated by PPP1R1A (expression increased by at least 2 fold upon PPP1R1A knockdown) revealed a subset of 16 replication-dependent histone genes (Supplementary Figure 2B).

Histone proteins are synthesized to package the newly replicated DNA during S phase. Interestingly, the normal replication-dependent histone mRNAs expressed during S phase end in a conserved stem-loop structure rather than a polyadenylated tail. However, these histone genes can be expressed as polyadenylated mRNAs in terminally differentiated cells and tissues or in growth arrested cells to allow for expression of histones outside of S phase [13]. Since polyA tailed transcripts were selected for RNA-seq analysis in control and PPP1R1A knockdown ES cells in our study, the population of histone mRNA that was sequenced and shown to be down-regulated by PPP1R1A should be polyadenylated. To confirm this, we performed RT-PCR to check the expression of both polyadenylated and total mRNAs of selected histone genes, *Hist1H2AC*, *Hist1H2BJ*, and *Hist1H4H*, in control and PPP1R1A knockdown cells. Indeed, we found that expression of the polyadenylated version of histone transcripts significantly increased while the total histone transcripts significantly decreased in PPP1R1A knockdown cells compared to those in control cells (Supplementary Figure 2C). These results indicate that PPP1R1A depletion blocked normal replication-dependent histone transcription while stimulating polyadenylated histone transcription in ES cells. The regulation of histone gene transcription by PPP1R1A further suggests that PPP1R1A regulates cell cycle in ES.

### PPP1R1A and IGF-1R inhibition synergize in limiting ES cell proliferation and migration

**Figure 3:**
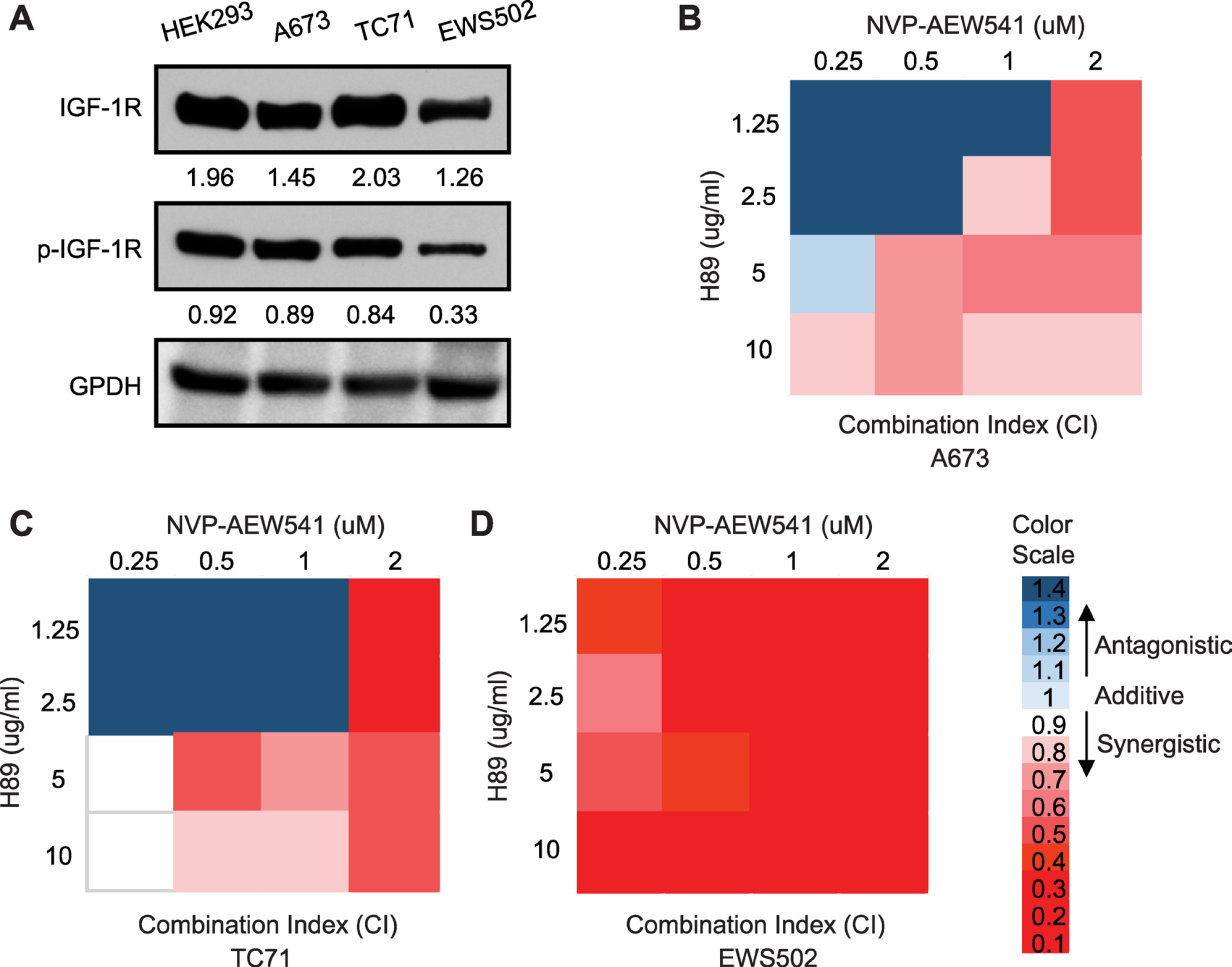
Inhibition of PPP1R1A and IGF-1R pathways synergizes in decreasing ES cell viability. (**A**) IGF-1R expression and phosphorylation levels in ES cells. (**B**–**D**) A673, TC71, and EWS502 cells were treated with increasing concentrations of H89, AEW541, or H89 together with AEW541 as indicated for 72 hours before cell proliferation was assessed by MTT assays. Bliss combination index (CI) was calculated and presented as heatmap. CI < 1 indicates synergism, = 1 additive effect, and > 1 antagonism.

Our above findings, including that PPP1R1A down-regulates cell cycle inhibitors p21^Cip1^ and p27^Kip1^, up-regulates Rb phosphorylation, and decreases replication-dependent histone gene transcription, all point to PPP1R1A as a cell cycle modulator in ES. Since PPP1R1A is specifically and highly expressed in ES [3], inhibition of PPP1R1A will specifically inhibit ES cell proliferation and therefore constitute a specific and tolerable therapeutic strategy to control ES. We previously utilized a small molecule inhibitor H89 which inhibits the PKA/PPP1R1A/PP1 pathway to treat ES cells *in vitro* and in ES orthotopic xenografts [3]. We found that H89 limited oncogenic transformation and migration of ES cells, and tumorigenesis and metastasis of ES xenograft tumors. Recently, cell cycle modulators, specifically CDK4/6 inhibitors, were shown to synergize with IGF-1R inhibitor in treating ES xenograft tumors [11]. To investigate whether PPP1R1A, as an ES specific cell cycle modulator, also has a synergistic or additive effect with IGF-1R inhibitor on ES cell viability, we first validated that IGF-1R signaling is active in ES cells by examining the expression and phosphorylation of IGF-1R in multiple ES cell lines (Figure 3A), and then treated ES cells with vehicle or increasing concentrations of H89, NVP-AEW541 (a small molecule IGF-1R inhibitor) (AEW541), or H89 together with AEW541, and carried out MTT cell proliferation analysis. Bliss combination index (CI) values were then calculated to assess the synergistic, additive, or antagonistic effects of the two drugs on cell viability [14]. Indeed, we found that these two compounds, in combination at various concentrations, are more effective than either of the single treatment alone, indicating a synergistic effect (CI < 1) (Figure 3B–3D). Interestingly, synergism between the two agents was much stronger when low concentrations of H89 were combined with high concentrations of AEW541, while additive and antagonistic effects were observed in A673 and TC71 cells when concentrations of both drugs were low. This is likely due to variation in drug-drug interaction in different cellular background which was previously reported [11, 15].

**Figure 4:**
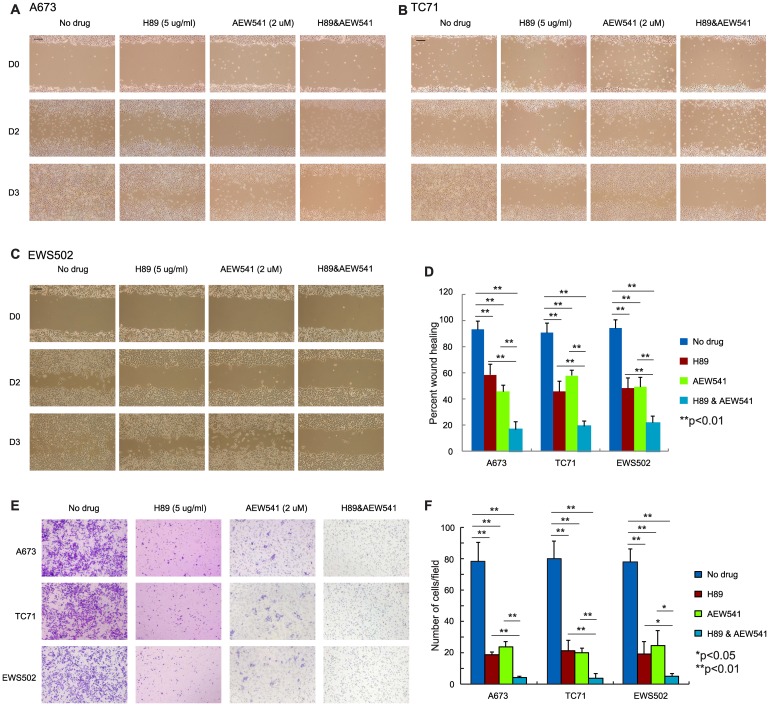
Simultaneous inhibition of PPP1R1A and IGF-1R pathways is more effective in limiting ES cell migration than single treatment. (**A**–**C**) A673 (A), TC71 (B), and EWS502 (C) ES cells treated with H89 in combination with AEW541 migrated much slower than the non-treated control or single agent treated cells. Scale bar equals 250 mm. (**D**) Quantification of wound healing assay results in three ES cell lines. ^**^ *p* < 0.001. (**E**) Boyden chamber transwell assay results showing that ES cell migration was significantly decreased when cells were treated with H89 and AEW541 compared with cells treated with or without H89 or AEW541 alone. (**F**) Quantification of the number of migrated cells per field in transwell assay in E. ^**^ *p* < 0.01, ^*^ *p* < 0.05.

We next investigated whether H89 and AEW541 combinatorial treatment also affected ES cell migration given that PPP1R1A and IGF-1R were both shown to play an important role in ES migration and metastasis [3, 16]. Wound healing and Boyden chamber assays were performed to evaluate the migratory ability of the ES cells treated with H89 alone, AEW541 alone, or H89 combined with AEW541. In wound healing assays, we observed that the cells treated with H89 or AEW541 alone migrated more slowly than control cells, and combination of H89 and AEW541 further slowed down the wound healing process compared to single agent (Figure 4A–4D). Since the growth inhibition induced by H89 and/or AEW541 (Supplementary Figure 3) may contribute to the impaired migration of the treated cells in wound healing assays which lasted for 72 hours, we performed Boyden chamber transwell assays with a 24 hour duration in which the effect of the two drugs on cell proliferation is minimum (Supplementary Figure 3). In Boyden chamber transwell assays, we found that cells treated with H89 or AEW541 alone had decreased translocation through a porous membrane compared to untreated cells, while treatment with H89 together with AEW541 further limited the ability of cells to migrate through the membrane (Figures 4E, 4F). Taken together, these results suggest that PPP1R1A inhibition combined with IGF-1R inhibition had synergistic/additive effects on ES cell viability and migration.

### Combination of PPP1R1A and IGF-1R inhibition reduced ES xenograft tumor growth and metastasis

**Figure 5:**
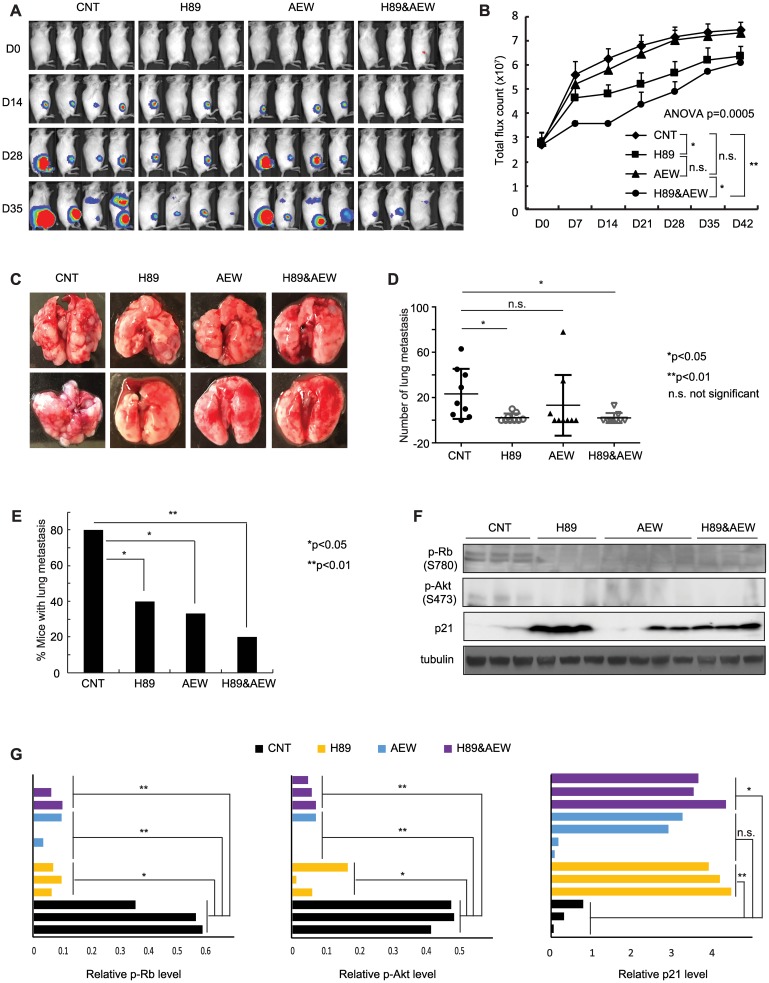
Combination of PPP1R1A and IGF-1R inhibition is more active in limiting ES tumor growth and metastasis than either individual treatment in an orthotopic xenograft mouse model. (**A**) and (**B**) *In vivo* xenograft studies measuring tumor growth in animals injected with luciferase expressing A673 cells and treated with vehicle or 8 mg/kg H89 or 50 mg/kg AEW541 or H89 together with AEW541 in intratibial injection mouse model. *n* = 10 for each group. ANOVA test *p =* 0.0005. ^*^post hoc test *p* < 0.05; ^**^ *p* < 0.01; n. s. not significant. (**C**) Representative images of lungs from the animals injected with A673 cells and treated with vehicle or H89 or AEW541 or H89 with AEW541 as indicated. (**D**) and (**E**) Graphs showing the number of metastatic nodules in each lung (D) and the percentage of mice with pulmonary lesions (E) in each indicated condition. ^*^Student’s *t*-test *p* < 0.05; n. s., not significant in (D). ^*^Two samples *Z* test for proportions *p* < 0.05, ^**^ *p* < 0.01 in (E). (**F**) and (**G**) p21^Cip1^ protein and phosphorylated Rb and Akt levels in tumors harvested from different treatment groups as indicated.

We next extended the H89 and AEW541 combination treatment to *in vivo* studies using an ES orthotopic xenograft mouse model. We have shown previously that H89 treatment at a dose of 10 mg/kg slowed down the development of primary tumors, although this result did not reach statistical significance because of the early termination of treatment due to side effects [3]. In this study, we lowered the dose of H89 to 8 mg/kg to reduce the side effects while maintaining the effectiveness of the drug by completing the entire planned treatment course. We found that H89 at this dosage significantly decreased primary tumor growth (*p* < 0.05, Figure 5A, 5B). For AEW541, a dose of 50 mg/kg that was reported to have less severe toxicity was utilized [17]. We found that AEW541 alone at this dosage had a mild effect on limiting primary tumor growth in this cell line-derived orthotopic xenograft mouse model, which is consistent with the observation in a recent report [11]. Importantly, the combination of the two drugs was more effective than either monotherapy in decreasing the primary tumor growth (analysis of variance (ANOVA) *p =* 0.0005, Figures 5A, 5B). Furthermore, the combination treatment significantly decreased the number of lung nodules (*p* < 0.05) and the percentage of mice with lung metastases (*p* < 0.01) compared to the vehicle treatment, and was more effective than H89 or AEW541 treatment alone in mitigating tumor metastasis (Figures 5A, 5C–5E). We evaluated target inhibition in mice sacrificed at the end of the treatment and found that Rb and Akt phosphorylation levels were markedly decreased in the H89, AEW541, and the combination groups as compared with the vehicle control. Conversely, p21^Cip1^ expression was drastically increased in these treatment groups compared to the control group (Figures 5F, 5G). These data suggest that combinatorial therapy of PPP1R1A and IGF-1R inhibition were more effective than single agent in reducing ES xenograft tumor growth and metastasis.

## DISCUSSION

We previously demonstrated that PPP1R1A is an important EWS/FLI target gene and plays a critical role in ES pathogenesis by promoting ES tumor growth and metastasis via the PKA/PPP1R1A/PP1 pathway [3]. In the current study, we define the role of PPP1R1A as an ES specific cell cycle modulator in G1 phase by down-regulating cell cycle inhibitors p21^Cip1^ and p27^Kip1^, activating Rb phosphorylation, and controlling histone gene expression (Figures 1, 2 and Supplementary Figures 1, 2), which have not been previously appreciated. More importantly, the discovery of the novel role of PPP1R1A in ES cell cycle modulation led us to test a rational combinatorial therapeutic strategy that is effective not only in limiting cell viability and tumor growth, but also cell migration and tumor metastasis (Figures 3–5). Metastasis at diagnosis is the most important adverse prognostic factor in ES. However, limited progress has been made in the treatment of metastatic ES. EWS-ETS fusion proteins have a central role in the pathogenesis including metastasis of ES. However, EWS/FLI is historically difficult to target. An alternative is to target EWS/FLI transcription co-factors and/or targets. Two phase I clinical trials separately investigating SP-2577, a lysine-specific histone demethylase 1 inhibitor, and TK216, a small molecule that interferes with EWS/FLI and RNA helicase A interaction, are ongoing in relapsed/refractory ES patients (NCT03600649 and NCT02657005). Since PPP1R1A is directly up-regulated by EWS/FLI and specifically highly expressed in ES but not the putative cell of origin, mesenchymal stem cells [3], our findings suggest a novel specific therapeutic strategy that is promising in enhancing therapeutic efficacy and improving outcome of patients with metastatic ES.

Cell cycle dysregulation is one of the hallmarks of cancer [18]. EWS/FLI was previously found to significantly up-regulate the expression levels of G1 cyclins, including cyclin D1 and E, and downregulate the two important cyclin-dependent kinase inhibitors for the G1/S transition, p21^Cip1^ and p27^Kip1^ [9, 10]. However, targeting the dysregulated cell cycle in ES is difficult due to the important role of these proteins in normal cells and the challenges of effectively targeting the EWS/FLI fusion protein. PPP1R1A, on the other hand, is specifically expressed in ES, making it an ideal specific therapeutic target. PP1 inhibitory subunits have previously been shown to regulate cell cycle progression through inhibition of PP1; PPP1R1B was found to regulate Cdk5 activity in neurons [19], whereas PPP1R2 can directly activate the Aurora A mitotic kinase and is required for normal mitotic progression [20, 21]. However, we believe this is the first report demonstrating a role of PPP1R1A in cell cycle modulation. Our data showed that the cell cycle arrest induced by PPP1R1A depletion can be rescued by T35D (Figure 2A), the constitutively active form which potently inhibits PP1, indicating that PPP1R1A mediated cell cycle control is at least in part through PP1 inhibition and post-translational modification in protein phosphorylation. In addition, we showed that PPP1R1A downregulates p21^Cip1^ and p27^Kip1^ transcription (RNA-seq) and protein abundance (western) (Figure 2C, 2D), suggesting a second mechanism by which PPP1R1A regulates the cell cycle. Nevertheless, the ultimate effect of these two mechanisms is the hyperphosphorylation of Rb protein and derepression of cell cycle.

p21^Cip1^ is a member of universal CDK inhibitors and plays crucial roles in the regulation of G1/S transition. p21^Cip1^ can induce differentiation of normal and transformed cells and suppress malignant cell growth [22]. p21^Cip1^ transcription can be directly upregulated by p53 via p53-responsive elements located in the p21^Cip1^ promoter [23]. A variety of other transcription factors, including Sp1, Sp3, C/EBP, and the STAT family [24], together with cofactors such as p300/CBP, can also regulate p21^Cip1^ transcription. In an electromobility shift assay, EWS/FLI was shown to bind to the ETS consensus sequences in the p21^Cip1^ promoter, suggesting that p21^Cip1^ transcription could also be regulated by EWS/FLI. It would be interesting to elucidate the mechanisms by which PPP1R1A regulates p21^Cip1^ transcription. p27^Kip1^ blocks G1/S transition in the cell cycle mainly through inhibition of CDK2 and cyclin A/E complex [25]. Transcription of p27^Kip1^ is regulated/activated by Forkhead box O proteins (FOXO4, FOXO3a, and FOXO1a) [26]. p27^Kip1^ expression level is also regulated by post-translational modification and epigenetic modification (methylation and acetylation) [27]. In ES cells, EWS/FLI depletion resulted in increased stability of p27^Kip1^ via decreased Skp2-mediated proteasome degradation [28]. Various factors including cytoplasmic sequestration, proto-oncogene serine/threonine protein kinase (PIM1), Akt phosphorylation, and 14-3-3 binding can suppress transcriptional activity of FOXO proteins and in turn suppress p27^Kip1^ transcription. Interestingly, we found that inhibition of PPP1R1A pathway by H89 decreased Akt phosphorylation (Figure 5E), suggesting a possible mechanism by which PPP1R1A regulates transcription of p27^Kip1^.

One interesting and unexpected finding in this report is the regulation of histone gene transcription by PPP1R1A. Histone protein synthesis is essential for the proper packaging of newly synthesized DNA into chromatin in S phase of the cell cycle. Unlike regular gene transcription, normal replication-dependent histone mRNA are not polyadenylated but end in a conserved 3′ stem-loop structure. This is due to the need of cell cycle dependent synthesis and degradation of histone transcripts; the stem-loop structure facilitates degradation while the polyA tailed transcripts are more stable [29]. However, when normal histone pre-mRNA processing is lost, such as in tumorigenesis, or terminal differentiation, histone mRNAs do get polyadenylated [30–33], suggesting an important physiological role of these polyA-tailed histone transcripts. We found that the level of normal histone transcripts decreased while that of polyadenylated ones increased upon PPP1R1A depletion (Supplementary Figure 2C), demonstrating that PPP1R1A is critical for cell cycle dependent normal histone gene transcription, and suggesting a compensatory mechanism which cancer cells utilize to cope with the loss of normal histone transcripts and to survive cell cycle arrest. It is also possible that PPP1R1A depletion induced terminal differentiation featured by polyadenylation of histone transcripts in ES cells. It was reported that CDK9, a substrate of PP1 [34], controls histone mRNA 3′-end processing, and CDK9 knockdown led to increased polyadenylation of histone mRNA [35]. Further investigation on whether CDK9 plays a role in PPP1R1A depletion mediated increase in polyadenylation of histone transcripts and whether these transcripts are related to the pathogenesis of ES is ongoing.

The IGF-1R/IGF pathway promotes cell-cycle progression at several phases, mainly at the G1/S transition, by increasing cyclin D1 and CDK4/6 gene expression, leading to Rb protein phosphorylation, release of E2F, and synthesis of cyclin E, which is mediated through the PI-3K/Akt and/or ERK axis [36]. In addition, IGF-1R can down-regulate the transcription of p27^Kip1^ or alter its processing and nuclear localization through a PI-3K/Akt and phosphatase and tensin homologous on chromosome 10 -dependent mechanism [36]. It was found that overexpression of IGF-1R was significantly linked to gain of a metastatic phenotype in synovial sarcoma, melanoma, and gastric cancer [37–39]. In ES, IGF-1R signaling is constitutively active and has been implicated in the tumorigenesis, growth, proliferation, and the development of metastatic disease [16, 40]. In several clinical trials of IGF-1R monoclonal antibodies for patients with relapsed ES, there has been a durable 10-15% response rate; IGF-1R inhibitors in upfront therapy for patients with metastatic ES have also been tested in a Phase 3 study [41–43]. Despite some durable responses, the overall results of these clinical trials shown limited efficacy of IGF-1R antibodies or inhibitors as a monotherapy, likely due to the lack of biomarkers to stratify patients and the development of treatment resistance in these patients. Thus, combination therapies that can enhance the efficacy of IGF-1R inhibitors are of particular interest to the ES clinical investigators. Recently, Guenther and colleagues found that IGF-1R overexpression was a resistance mechanism to CDK4/6 inhibitors; a CDK4/6 inhibitor and an IGF-1R inhibitor were synergistic *in vitro* against ES cells and the combination of these two inhibitors were more effective than single regimen in ES tumor control in mouse models [11]. In the current study, we found that combinatorial therapy of PPP1R1A and IGF-1R inhibitors is superior to single treatment not only in limiting tumor growth but also lung metastasis (Figure 5) which has never been reported in prior studies. We speculate that the superior effect of combination is due to the simultaneous inhibition of the PPP1R1A and IGF-1R pathways which are both important cascades in ES cell cycle progression and cell migration/invasion.

In conclusion, we demonstrate that PPP1R1A is an ES specific cell cycle modulator which promotes cell cycle progression from G1 to S phase by negatively regulating cell cycle inhibitors p21^Cip1^ and p27^Kip1^ and promoting normal transcription of replication-dependent histone genes and that the combinatorial inhibition of PPP1R1A pathway with an IGF-1R inhibitor has synergistic/additive effects on ES cell and tumor growth and dissemination. Our findings strongly suggest further investigation of this combinatorial therapy in patients with primary and metastatic ES.

## MATERIALS AND METHODS

### Cell lines and chemicals

A673 cells were obtained from ATCC (Manassas, VA, USA) and maintained as previously described [44]. TC71 and EWS502 were obtained from Children’s Oncology Group Cell Line and Xenograft Repository (Lubbock, TX, USA) and cultured as previously described [45]. Short tandem repeat profile of cell lines is shown in Supplementary File 2. ES cell lines were treated with the IGF-1R inhibitor NVP-AEW541 kindly provided by Novartis Oncology (Cambridge, MA, USA) and the PKA inhibitor H89 obtained from MilliporeSigma (B1427) (Burlington, MA, USA).

### Growth curve and cell cycle analysis

Cell growth was assessed by 3T5 assay as we have previously described [44]. For cell cycle analysis, ES cells were fixed with 70% ethanol and treated with 5 mg/ml RNase for 30 min. After staining with 50 mM of propidium iodide (PI), the cells were subjected to flow cytometry analysis with MACSquant (Miltenyi Biotec, Somerville, MA, USA).

### Cell viability and migration assays

ES cells were treated with various concentrations of H89 and/or NVP-AEW541 for 24 or 72 hours and MTT assays (Cayman Chemical, Ann Arbor, MI, USA) were performed as we have previously described [44]. Boyden chamber and wound healing assays were performed as previously described [46, 47].

### Synergy studies

Synergy was assessed by Chou-Talalay Combination Index using COMPUSYN as previously described [14].

### Constructs

RNA interference constructs for control (iLuc) and PPP1R1A (iR1A-1, -3) were generated and used as previously described [3, 48]. The PPP1R1A knockout construct (R1A-KO3) was generated by cloning the CRISPR guide RNA against PPP1R1A into the lentiCRISPRv2 vector from Feng Zhang laboratory at the Massachusetts Institute of Technology (Cambridge, MA, USA) (Addgene plasmid #52961) [49]. Guide sequences were designed using the Broad Institute sgRNA designer tool (https://portals.broadinstitute.org/gpp/public/analysis-tools/sgrna-design) and are shown in Supplementary File 3. For CRISPR-Cas9 knockout of PPP1R1A, following virus infection of the gRNAs, polyclonal cell populations were prepared for further analysis by growth in the selective media. PPP1R1A T35D was generated as previously described [3].

### Quantitative reverse transcriptase polymerase chain reaction (qRT-PCR)

Total RNA was extracted by using an RNeasy mini kit (Qiagen, Hilden, Germany). Reverse-transcriptase polymerase chain reaction was performed using iScript SYBR green RT-PCR kit (Bio-Rad Laboratories, Hercules, CA, USA). Primer sequences are shown in Supplementary File 3.

### RNA-sequencing data analysis

The PPP1R1A RNA-seq data from the Sequence Read Archive database (accession number SRP089716) [3] were used for identification of PPP1R1A regulated genes. Reads were aligned with Tophat version 2.1.0 to hg19 genome build. Aligned BAM files were assessed for differential gene expression (defined as: false discovery rate ≤ 0.05 and |log2 fold change| ≥ 1.3) comparing iLuc and iR1A-1 groups using cufflinks version 2.2.1. DAVID functional annotation clustering algorithm (david. abcc. ncifcrf. gov) was used to identify functional classes that were enriched in the PPP1R1A regulated gene list.

### Western immunoblotting

Immunoblotting was performed as we have previously described [50]. Anti-PPP1R1A (ab40877) was purchased from Abcam Inc. (Cambridge, MA, USA). Anti-tubulin (sc-23948) was from Santa Cruz Biotechnology, Inc. (Dallas, TX, USA). Cell cycle regulation antibody sampler kit (9932) and Rb antibody sampler kit (9969) were from Cell Signaling Technology (Danvers, MA, USA). Quantification of band intensity was performed by ImageJ (https://imagej.nih.gov/ij).

### Animal studies

All animal studies were performed in accordance with protocols approved by the New York Medical College Institutional Animal Care and Use Committee (NYMC, 22-2-0618H), Valhalla, NY, USA. Four to six weeks old female NOD-SCID mice (Charles River Laboratories, Wilmington, MA, USA) were injected intra-tibially with 1 × 10^5^ of luciferase expressing A673 cells re-suspended in growth factor reduced Matrigel matrix (BD Biosciences, San Jose, CA, USA). Mice were grouped randomly and treated with vehicle or H89 or NVP-AEW541 or H89 together with NVP-AEW541 24 hours after tumor cell injection. For H89 treatment, 8 mg/kg of H89 in 5% dimethyl sulfoxide or vehicle was injected intraperitoneally twice a week for 4 weeks. NVP-AEW541 (50 mg/kg) was given twice a day for 2 weeks via gavage. *N* = 10 per group. Tumor growth was monitored by imaging using Xenogen IVIS 100 imaging system weekly after injection as we have previously described [51]. Mice were sacrificed 6 weeks after injection or tumor size reaches 2 cm^3^ and the tumors and lungs were harvested for evaluation of protein expression and lung metastases as we have previously described [3].

### Statistical analyses

Statistical differences were determined using Student *t* test for paired data, or two samples Z test for proportions (one-tailed) where percentage of mice with lung metastasis was analyzed. Bonferroni–Holm post hoc test after one-way ANOVA was used for data sets of multiple comparisons. All data are presented as the mean±SD of at least three independent experiments except where stated. Error bars represent standard deviation, unless otherwise stated. The threshold for statistical significance is *p* < 0.05, unless otherwise specified.

## SUPPLEMENTARY MATERIALS





## Abbreviations

ES: Ewing Sarcoma
PPPIR1A: protein phosphatase 1 regulatory subunit 1A
Rb: retinoblastoma
mRNA: messenger RNA
IGF-IR: insulin-like growth factor 1 receptor
ETS: E26 transformation-specific
PP1: protein phosphatase 1
PKA: protein kinase A
CDK: cyclin-dependent kinase
CIP/KIP: CDK interacting protein/Kinase inhibitory protein
CRISPR: Clustered Regularly Interspaced Short Palindromic Repeats
DAVID: Database for Annotation, Visualization and Integrated Discovery
MTT: 3-(4,5-dimethylthiazol-2-yl)-2,5-diphenyltetrazolium bromide
CI: combination index
ANOVA: analysis of variance
FOXO: Forkhead box O proteins
AKT: protein kinase B
PI: propidium iodide
qRT-PCR: quantitative reverse transcriptase polymerase chain reaction

## References

[1] Ladenstein R, Pötschger U, Le Deley MC, Whelan J, Paulussen M, Oberlin O, van den Berg H, Dirksen U, Hjorth L, Michon J, Lewis I, Craft A, Jürgens H Primary disseminated multifocal Ewing sarcoma: results of the Euro-EWING 99 trial. J Clin Oncol. 2010; 28:3284–91. 10.1200/JCO.2009.22.9864. 20547982

[2] Delattre O, Zucman J, Plougastel B, Desmaze C, Melot T, Peter M, Kovar H, Joubert I, de Jong P, Rouleau G, Aurias A, Thomas G Gene fusion with an ETS DNA-binding domain caused by chromosome translocation in human tumours. Nature. 1992; 359:162–65. 10.1038/359162a0. 1522903

[3] Luo W, Xu C, Ayello J, Dela Cruz F, Rosenblum JM, Lessnick SL, Cairo MS Protein phosphatase 1 regulatory subunit 1A in ewing sarcoma tumorigenesis and metastasis. Oncogene. 2018; 37:798–809. 10.1038/onc.2017.378. 29059150

[4] Malumbres M, Barbacid M Cell cycle, CDKs and cancer: a changing paradigm. Nat Rev Cancer. 2009; 9:153–66. 10.1038/nrc2602. 19238148

[5] Abbas T, Dutta A p21 in cancer: intricate networks and multiple activities. Nat Rev Cancer. 2009; 9:400–14. 10.1038/nrc2657. 19440234 PMC2722839

[6] Kennedy AL, Vallurupalli M, Chen L, Crompton B, Cowley G, Vazquez F, Weir BA, Tsherniak A, Parasuraman S, Kim S, Alexe G, Stegmaier K Functional, chemical genomic, and super-enhancer screening identify sensitivity to cyclin D1/CDK4 pathway inhibition in Ewing sarcoma. Oncotarget. 2015; 6:30178–93. 10.18632/oncotarget.4903. 26337082 PMC4745789

[7] Kowalewski AA, Randall RL, Lessnick SL Cell Cycle Deregulation in Ewing’s Sarcoma Pathogenesis. Sarcoma. 2011; 2011:598704. 10.1155/2011/598704. 21052502 PMC2968116

[8] Maitra A, Roberts H, Weinberg AG, Geradts J Aberrant expression of tumor suppressor proteins in the Ewing family of tumors. Arch Pathol Lab Med. 2001; 125:1207–12. 11520274 10.5858/2001-125-1207-AEOTSP

[9] Matsunobu T, Tanaka K, Matsumoto Y, Nakatani F, Sakimura R, Hanada M, Li X, Oda Y, Naruse I, Hoshino H, Tsuneyoshi M, Miura H, Iwamoto Y The prognostic and therapeutic relevance of p27kip1 in Ewing’s family tumors. Clin Cancer Res. 2004; 10:1003–12. 10.1158/1078-0432.CCR-0788-3. 14871979

[10] Nakatani F, Tanaka K, Sakimura R, Matsumoto Y, Matsunobu T, Li X, Hanada M, Okada T, Iwamoto Y Identification of p21WAF1/CIP1 as a direct target of EWS-Fli1 oncogenic fusion protein. J Biol Chem. 2003; 278:15105–15. 10.1074/jbc.M211470200. 12560328

[11] Guenther LM, Dharia NV, Ross L, Conway A, Robichaud AL, Catlett JL 2nd, Wechsler CS, Frank ES, Goodale A, Church AJ, Tseng YY, Guha R, McKnight CG, et al A Combination CDK4/6 and IGF1R Inhibitor Strategy for Ewing Sarcoma. Clin Cancer Res. 2019; 25:1343–57. 10.1158/1078-0432.CCR-18-0372. 30397176 PMC6498855

[12] Ma D, Zhou P, Harbour JW Distinct mechanisms for regulating the tumor suppressor and antiapoptotic functions of Rb. J Biol Chem. 2003; 278:19358–66. 10.1074/jbc.M301761200. 12646568

[13] Lyons SM, Cunningham CH, Welch JD, Groh B, Guo AY, Wei B, Whitfield ML, Xiong Y, Marzluff WF A subset of replication-dependent histone mRNAs are expressed as polyadenylated RNAs in terminally differentiated tissues. Nucleic Acids Res. 2016; 44:9190–205. 10.1093/nar/gkw620. 27402160 PMC5100578

[14] Chou TC Drug combination studies and their synergy quantification using the Chou-Talalay method. Cancer Res. 2010; 70:440–46. 10.1158/0008-5472.CAN-09-1947. 20068163

[15] Zhuo R, Kosak KM, Sankar S, Wiles ET, Sun Y, Zhang J, Ayello J, Prestwich GD, Shami PJ, Cairo MS, Lessnick SL, Luo W Targeting Glutathione S-transferase M4 in Ewing sarcoma. Front Pediatr. 2014; 2:83. 10.3389/fped.2014.00083. 25147782 PMC4123608

[16] Scotlandi K, Benini S, Nanni P, Lollini PL, Nicoletti G, Landuzzi L, Serra M, Manara MC, Picci P, Baldini N Blockage of insulin-like growth factor-I receptor inhibits the growth of Ewing’s sarcoma in athymic mice. Cancer Res. 1998; 58:4127–31. 9751624

[17] Manara MC, Landuzzi L, Nanni P, Nicoletti G, Zambelli D, Lollini PL, Nanni C, Hofmann F, García-Echeverría C, Picci P, Scotlandi K Preclinical *in vivo* study of new insulin-like growth factor-I receptor—specific inhibitor in Ewing’s sarcoma. Clin Cancer Res. 2007; 13:1322–30. 10.1158/1078-0432.CCR-06-1518. 17317844

[18] Hanahan D, Weinberg RA Hallmarks of cancer: the next generation. Cell. 2011; 144:646–74. 10.1016/j.cell.2011.02.013. 21376230

[19] Bibb JA, Snyder GL, Nishi A, Yan Z, Meijer L, Fienberg AA, Tsai LH, Kwon YT, Girault JA, Czernik AJ, Huganir RL, Hemmings HC Jr, Nairn AC, Greengard P Phosphorylation of DARPP-32 by Cdk5 modulates dopamine signalling in neurons. Nature. 1999; 402:669–71. 10.1038/45251. 10604473

[20] Satinover DL, Leach CA, Stukenberg PT, Brautigan DL Activation of Aurora-A kinase by protein phosphatase inhibitor-2, a bifunctional signaling protein. Proc Natl Acad Sci USA. 2004; 101:8625–30. 10.1073/pnas.0402966101. 15173575 PMC423245

[21] Wang W, Stukenberg PT, Brautigan DL Phosphatase inhibitor-2 balances protein phosphatase 1 and aurora B kinase for chromosome segregation and cytokinesis in human retinal epithelial cells. Mol Biol Cell. 2008; 19:4852–62. 10.1091/mbc.e08-05-0460. 18716057 PMC2575180

[22] Gartel AL, Serfas MS, Tyner AL p21—negative regulator of the cell cycle. Proc Soc Exp Biol Med. 1996; 213:138–49. 10.3181/00379727-213-44046. 8931660

[23] El-Deiry WS, Tokino T, Velculescu VE, Levy DB, Parsons R, Trent JM, Lin D, Mercer WE, Kinzler KW, Vogelstein B WAF1, a potential mediator of p53 tumor suppression. Cell. 1993; 75:817–25. 10.1016/0092-8674(93)90500-P. 8242752

[24] Gartel AL, Tyner AL Transcriptional regulation of the p21((WAF1/CIP1)) gene. Exp Cell Res. 1999; 246:280–89. 10.1006/excr.1998.4319. 9925742

[25] Polyak K, Kato JY, Solomon MJ, Sherr CJ, Massague J, Roberts JM, Koff A p27Kip1, a cyclin-Cdk inhibitor, links transforming growth factor-beta and contact inhibition to cell cycle arrest. Genes Dev. 1994; 8:9–22. 10.1101/gad.8.1.9. 8288131

[26] Bertoli C, Copetti T, Lam EW, Demarchi F, Schneider C Calpain small-1 modulates Akt/FoxO3A signaling and apoptosis through PP2A. Oncogene. 2009; 28:721–33. 10.1038/onc.2008.425. 19029949

[27] Abbastabar M, Kheyrollah M, Azizian K, Bagherlou N, Tehrani SS, Maniati M, Karimian A Multiple functions of p27 in cell cycle, apoptosis, epigenetic modification and transcriptional regulation for the control of cell growth: A double-edged sword protein. DNA Repair (Amst). 2018; 69:63–72. 10.1016/j.dnarep.2018.07.008. 30075372

[28] Matsunobu T, Tanaka K, Nakamura T, Nakatani F, Sakimura R, Hanada M, Li X, Okada T, Oda Y, Tsuneyoshi M, Iwamoto Y The possible role of EWS-Fli1 in evasion of senescence in Ewing family tumors. Cancer Res. 2006; 66:803–11. 10.1158/0008-5472.CAN-05-1972. 16424012

[29] Pandey NB, Marzluff WF The stem-loop structure at the 3′ end of histone mRNA is necessary and sufficient for regulation of histone mRNA stability. Mol Cell Biol. 1987; 7:4557–59. 10.1128/MCB.7.12.4557. 3437896 PMC368142

[30] Kirsh AL, Groudine M, Challoner PB Polyadenylation and U7 snRNP-mediated cleavage: alternative modes of RNA 3′ processing in two avian histone H1 genes. Genes Dev. 1989; 3:2172–79. 10.1101/gad.3.12b.2172. 2576416

[31] Martinez I, Wang J, Hobson KF, Ferris RL, Khan SA Identification of differentially expressed genes in HPV-positive and HPV-negative oropharyngeal squamous cell carcinomas. Eur J Cancer. 2007; 43:415–32. 10.1016/j.ejca.2006.09.001. 17079134 PMC1847595

[32] Zhao H, Langerød A, Ji Y, Nowels KW, Nesland JM, Tibshirani R, Bukholm IK, Kåresen R, Botstein D, Børresen-Dale AL, Jeffrey SS Different gene expression patterns in invasive lobular and ductal carcinomas of the breast. Mol Biol Cell. 2004; 15:2523–36. 10.1091/mbc.e03-11-0786. 15034139 PMC420079

[33] Kari V, Karpiuk O, Tieg B, Kriegs M, Dikomey E, Krebber H, Begus-Nahrmann Y, Johnsen SA A subset of histone H2B genes produces polyadenylated mRNAs under a variety of cellular conditions. PLoS One. 2013; 8:e63745. 10.1371/journal.pone.0063745. 23717473 PMC3661734

[34] Ammosova T, Obukhov Y, Kotelkin A, Breuer D, Beullens M, Gordeuk VR, Bollen M, Nekhai S Protein phosphatase-1 activates CDK9 by dephosphorylating Ser175. PLoS One. 2011; 6:e18985. 10.1371/journal.pone.0018985. 21533037 PMC3080879

[35] Pirngruber J, Shchebet A, Schreiber L, Shema E, Minsky N, Chapman RD, Eick D, Aylon Y, Oren M, Johnsen SA CDK9 directs H2B monoubiquitination and controls replication-dependent histone mRNA 3′-end processing. EMBO Rep. 2009; 10:894–900. 10.1038/embor.2009.108. 19575011 PMC2726677

[36] Samani AA, Yakar S, LeRoith D, Brodt P The role of the IGF system in cancer growth and metastasis: overview and recent insights. Endocr Rev. 2007; 28:20–47. 10.1210/er.2006-0001. 16931767

[37] All-Ericsson C, Girnita L, Seregard S, Bartolazzi A, Jager MJ, Larsson O Insulin-like growth factor-1 receptor in uveal melanoma: a predictor for metastatic disease and a potential therapeutic target. Invest Ophthalmol Vis Sci. 2002; 43:1–8. 11773005

[38] Jiang Y, Wang L, Gong W, Wei D, Le X, Yao J, Ajani J, Abbruzzese JL, Huang S, Xie K A high expression level of insulin-like growth factor I receptor is associated with increased expression of transcription factor Sp1 and regional lymph node metastasis of human gastric cancer. Clin Exp Metastasis. 2004; 21:755–64. 10.1007/s10585-005-1198-2. 16035620

[39] Xie Y, Skytting B, Nilsson G, Brodin B, Larsson O Expression of insulin-like growth factor-1 receptor in synovial sarcoma: association with an aggressive phenotype. Cancer Res. 1999; 59:3588–91. 10446966

[40] Olmos D, Martins AS, Jones RL, Alam S, Scurr M, Judson IR Targeting the Insulin-Like Growth Factor 1 Receptor in Ewing’s Sarcoma: reality and Expectations. Sarcoma. 2011; 2011:402508. 10.1155/2011/402508. 21647361 PMC3103989

[41] Juergens H, Daw NC, Geoerger B, Ferrari S, Villarroel M, Aerts I, Whelan J, Dirksen U, Hixon ML, Yin D, Wang T, Green S, Paccagnella L, Gualberto A Preliminary efficacy of the anti-insulin-like growth factor type 1 receptor antibody figitumumab in patients with refractory Ewing sarcoma. J Clin Oncol. 2011; 29:4534–40. 10.1200/JCO.2010.33.0670. 22025154 PMC3236653

[42] Pappo AS, Patel SR, Crowley J, Reinke DK, Kuenkele KP, Chawla SP, Toner GC, Maki RG, Meyers PA, Chugh R, Ganjoo KN, Schuetze SM, Juergens H, et al R1507, a monoclonal antibody to the insulin-like growth factor 1 receptor, in patients with recurrent or refractory Ewing sarcoma family of tumors: results of a phase II Sarcoma Alliance for Research through Collaboration study. J Clin Oncol. 2011; 29:4541–47. 10.1200/JCO.2010.34.0000. 22025149 PMC3236654

[43] Tap WD, Demetri G, Barnette P, Desai J, Kavan P, Tozer R, Benedetto PW, Friberg G, Deng H, McCaffery I, Leitch I, Badola S, Chang S, et al Phase II study of ganitumab, a fully human anti-type-1 insulin-like growth factor receptor antibody, in patients with metastatic Ewing family tumors or desmoplastic small round cell tumors. J Clin Oncol. 2012; 30:1849–56. 10.1200/JCO.2011.37.2359. 22508822

[44] Luo W, Gangwal K, Sankar S, Boucher KM, Thomas D, Lessnick SL GSTM4 is a microsatellite-containing EWS/FLI target involved in Ewing’s sarcoma oncogenesis and therapeutic resistance. Oncogene. 2009; 28:4126–32. 10.1038/onc.2009.262. 19718047

[45] Lessnick SL, Dacwag CS, Golub TR The Ewing’s sarcoma oncoprotein EWS/FLI induces a p53-dependent growth arrest in primary human fibroblasts. Cancer Cell. 2002; 1:393–401. 10.1016/S1535-6108(02)00056-9. 12086853

[46] Chaturvedi A, Hoffman LM, Welm AL, Lessnick SL, Beckerle MC The EWS/FLI Oncogene Drives Changes in Cellular Morphology, Adhesion, and Migration in Ewing Sarcoma. Genes Cancer. 2012; 3:102–16. 10.1177/1947601912457024. 23050043 PMC3463921

[47] Wiles ET, Bell R, Thomas D, Beckerle M, Lessnick SL ZEB2 Represses the Epithelial Phenotype and Facilitates Metastasis in Ewing Sarcoma. Genes Cancer. 2013; 4:486–500. 10.1177/1947601913506115. 24386509 PMC3877663

[48] Smith R, Owen LA, Trem DJ, Wong JS, Whangbo JS, Golub TR, Lessnick SL Expression profiling of EWS/FLI identifies NKX2.2 as a critical target gene in Ewing’s sarcoma. Cancer Cell. 2006; 9:405–16. 10.1016/j.ccr.2006.04.004. 16697960

[49] Sanjana NE, Shalem O, Zhang F Improved vectors and genome-wide libraries for CRISPR screening. Nat Methods. 2014; 11:783–84. 10.1038/nmeth.3047. 25075903 PMC4486245

[50] Luo W, Peterson A, Garcia BA, Coombs G, Kofahl B, Heinrich R, Shabanowitz J, Hunt DF, Yost HJ, Virshup DM Protein phosphatase 1 regulates assembly and function of the beta-catenin degradation complex. EMBO J. 2007; 26:1511–21. 10.1038/sj.emboj.7601607. 17318175 PMC1829374

[51] Chu Y, Hochberg J, Yahr A, Ayello J, van de Ven C, Barth M, Czuczman M, Cairo MS Targeting CD20+ Aggressive B-cell Non-Hodgkin Lymphoma by Anti-CD20 CAR mRNA-Modified Expanded Natural Killer Cells *In Vitro* and in NSG Mice. Cancer Immunol Res. 2015; 3:333–44. 10.1158/2326-6066.CIR-14-0114. 25492700

