# Supplementary material for: Protein phosphatase 1 regulatory subunit 1A regulates cell cycle progression in Ewing sarcoma
Source: Oncotarget. 2020 May 12;11(19):1691–704. doi: 10.18632/oncotarget.27571 (PMC7233808; doi:10.18632/oncotarget.27571)
Supplement: Supplementary file 1 [file oncotarget-11-1691-s001.pdf]

Protein phosphatase 1 regulatory subunit 1A regulates cell cycle progression in ewing sarcoma

SUPPLEMENTARY MATERIALS

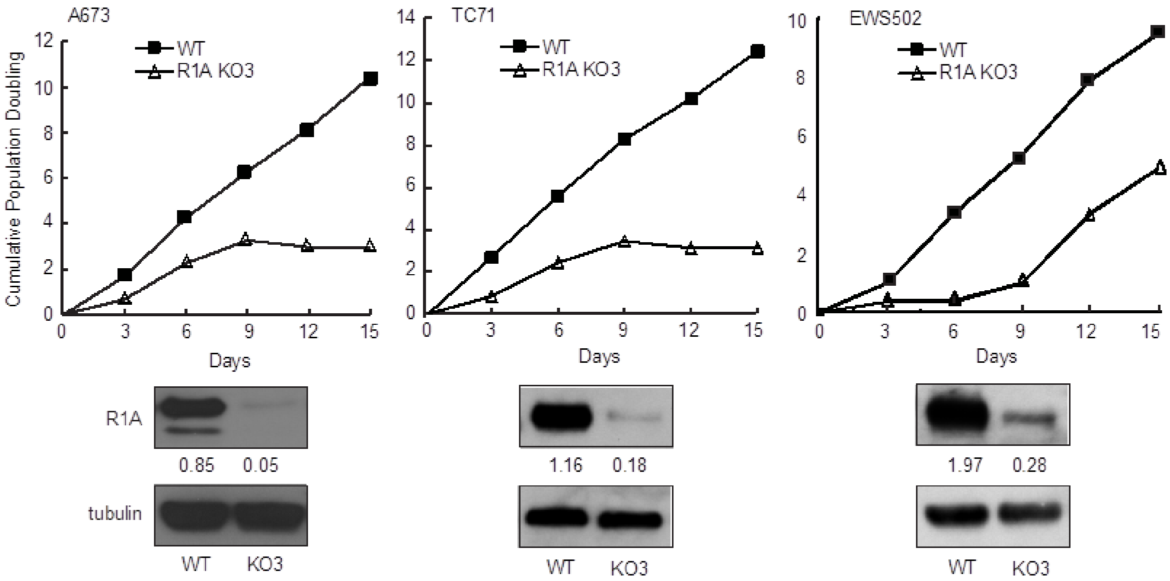

Supplementary Figure 1: CRISPR-Cas9 knockout of PPP1R1A leads to decrease in cell growth rate in multiple ES cell lines. Cumulative population doubling rate of wildtype (WT) or PPP1R1A knockout (R1A KO3) cells (*upper*) and PPP1R1A protein levels in these cells (*lower*) are shown.

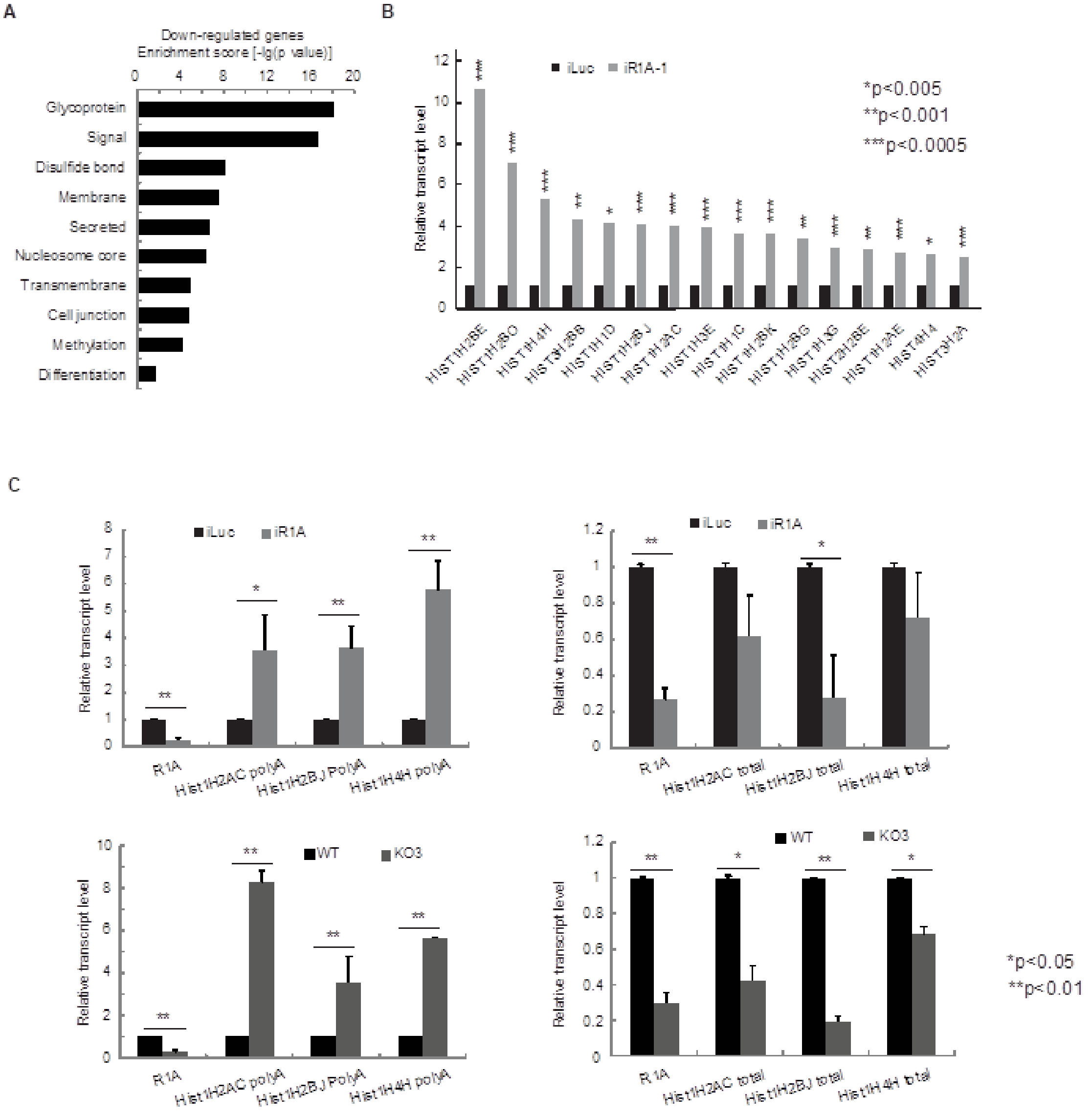

Supplementary Figure 2: PPP1R1A regulates replication-dependent histone gene transcription. (**A**) DAVID annotation analysis shows that nucleosome core is among the significantly enriched terms in PPP1R1A downregulated genes. (**B**) Transcriptional profiling of a subset of histone genes extracted from results of RNA-seq analysis in control (iLuc) and PPP1R1A (iR1A-1) knockdown cells showing increased transcript levels in PPP1R1A knockdown cells. ***multiple testing adjusted $p < 0.0005$, $^{**}p < 0.001$, $^{*}p < 0.005$. (**C**) qRT-PCR validation of PPP1R1A downregulation of polyadenylated (left), and upregulation of total (right), transcripts of select histone genes using RNA from control or PPP1R1A knockdown (upper) or CRISPR-Cas9 knockout (lower) ES cells. $^{*}p < 0.05$, $^{**}p < 0.01$.

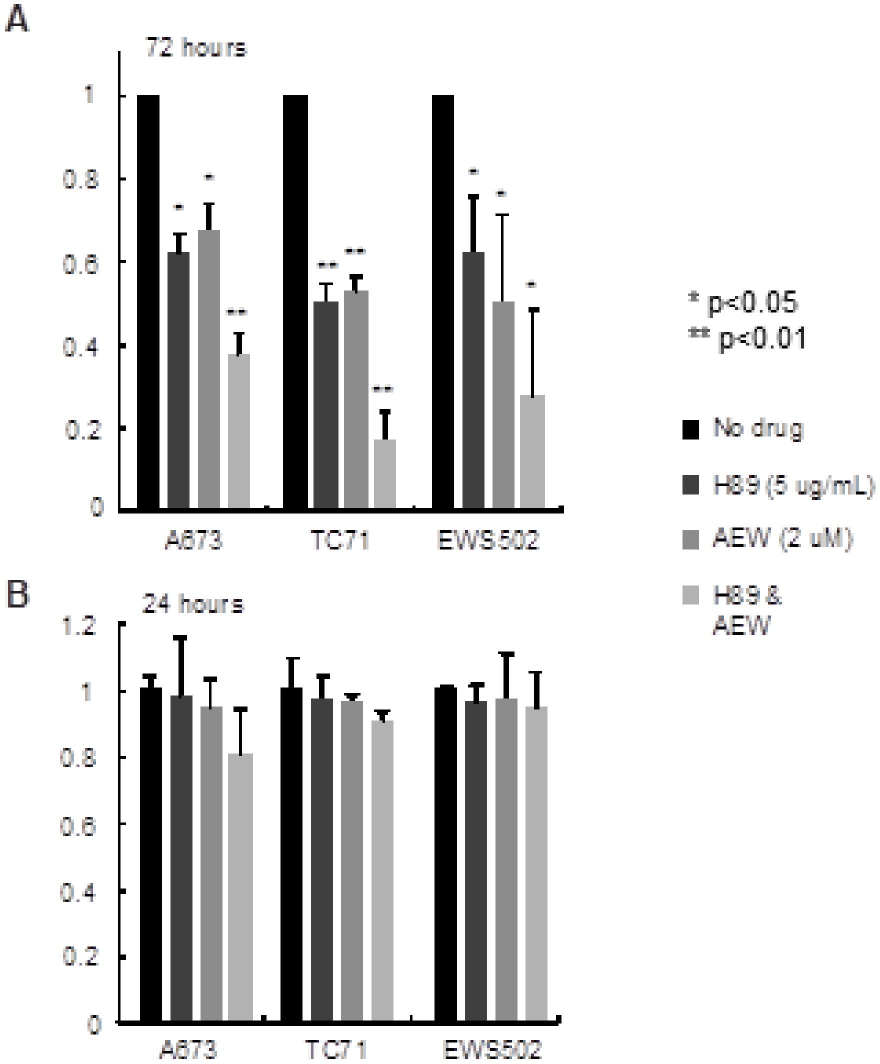

Supplementary Figure 3: ES cell viability after H89 and/or AEW541 treatment. A673, TC71, and EWS502 cells were treated with 5 ug/mL of H89 and/or 2 uM of AEW541 for 72 (**A**) and 24 (**B**) hours before MTT cell proliferation assays were carried out.

Supplementary File 1: Average intensity of western blotting bands in three repeats in Figure 2C.
*p<0.05 **p<0.01 compared to empty/iLuc. See Supplementary File 1

Supplementary File 2: Short random repeat profiles for ES cells

Cell line	STR profile
A673	Amelogenin: X
	CSF1PO: 11,12
	D13S317: 8,13
	D16S539: 11
	D5S818: 11,12
	D7S820: 10,12
	THO1: 9.3
	TPOX: 8
	vWA: 15,18
TC71	Amelogenin: X,Y (Cosmic-CLP; COG; PubMed=24312454)
	X (DSMZ; PubMed=25877200)
	CSF1PO: 10,11
	D13S317: 11,12
	D16S539: 11,14
	D18S51: 12,15
	D19S433: 14,16.2
	D21S11: 29,30
	D2S1338: 20,24
	D3S1358: 15,17
	D5S818: 10
	D7S820: 10
	D8S1179: 10,12
	FGA: 24,26
	Penta D: 11,14
	Penta E: 12
	TH01: 9.3
	TPOX: 8,9
	vWA: 17 (COG; DSMZ; PubMed=24312454; PubMed=25877200)
	17,19 (Cosmic-CLP)
EWS502	N/A

Supplementary File 3: gRNA sequences targeting PPP1R1A and RT-PCR primers

R1AKO1F:	5’-CACCGACCGTGAACTGGATCTTTCGGGG-3’
R1AKO1R:	5’-AAACCCCCGAAAGATCCAGTTCACGGTC-3’
R1AKO2F:	5’-CACCGAGGCGGCGGAGCAGATTCGG-3’
R1AKO2R:	5’-AAACCCGAATCTGCTCCGCCGCCTC-3’
R1AKO3F:	5’-CACCGGGAGCCGCACCTTGACCCCG-3’
R1AKO3R:	5’-AAACCGGGGTCAAGGTGCGGCTCCC-3’
HIST1H2AC Total F	5'-GACGAGGAGCTCAACAAACTG-3'
HIST1H2AC Total R	5'-ACCTGTCAAATCACTTGCCC-3'
HIST1H2AC PolyA F	5'-CCTGTCCACTGTTGGTAGGC-3'
HIST1H2AC PolyA R	5'-TTCACTTACCACCATTCCAGC-3'
HIST1H2BJ Total F	5'-GTGTACAAGGTTCTGAAGCAG-3'
HIST1H2BJ Total R	5'-CTGGAGGTGATGGTCGAGC-3'
HIST1H2BJ PolyA F	5'-CGCTCGACCATCACCTCCA-3'
HIST1H2BJ PolyA R	5'-CAGGTCACTAGTGTAGATGC-3'
HIST1H4H Total F	5’-GAGGAGACTCGTGGTGTTC-3’
HIST1H4H Total R	5’-GAGTGCAGCAAGCAGGAGC-3’
HIST1H4H PolyA F	5’-GCTCCTGCTTGCTGCACTC-3’
HIST1H4H PolyA R	5’-CTTCTCAGATCCATCAGGAG-3’
